# The effect of contextual interference on transfer in motor learning - a systematic review and meta-analysis

**DOI:** 10.3389/fpsyg.2024.1377122

**Published:** 2024-08-14

**Authors:** Stanisław H. Czyż, Aleksandra M. Wójcik, Petra Solarská

**Affiliations:** ^1^Faculty of Physical Education and Sports, Wroclaw University of Health and Sport Sciences, Wroclaw, Poland; ^2^Faculty of Sport Studies, Masaryk University, Brno, Czechia; ^3^Physical Activity, Sport and Recreation (PhASRec), Faculty of Health Sciences, North-West University, Potchefstroom, South Africa

**Keywords:** contextual interference, practice schedule, random practice, blocked practice, transfer, motor learning

## Abstract

Since the initial study on contextual interference (CI) in 1966, research has explored how practice schedules impact retention and transfer. Apart from support from scientists and practitioners, the CI effect has also faced skepticism. Therefore, we aimed to review the existing literature on the CI effect and determine how it affects transfer in laboratory and applied settings and in different age groups. We found 1,287 articles in the following databases: Scopus, EBSCO, Web of Science, ScienceDirect, supplemented by the Google Scholar search engine and manual search. Of 300 fully screened articles, 42 studies were included in the systematic review and 34 in the quantitative analysis (meta-analysis). The overall CI effect on transfer in motor learning was medium (SMD = 0.55), favoring random practice. Random practice was favored in the laboratory and applied settings. However, in laboratory studies, the medium effect size was statistically significant (SMD = 0.75), whereas, in applied studies, the effect size was small and statistically non-significant (SMD = 0.34). Age group analysis turned out to be significant only in adults and older adults. In both, the random practice was favored. In adults, the effect was medium (SMD = 0.54), whereas in older adults was large (SMD = 1.28). In young participants, the effect size was negligible (SMD = 0.12).

**Systematic review registration:** https://clinicaltrials.gov/, identifier CRD42021228267.

## Introduction

The very first meta-analysis on contextual interference (CI) in motor learning was published in 2004 by Brady (2004). Brady asked how CI affects retention and transfer, i.e., two processes which, according to Battig (1966, 1972), are altered by CI level. Battig noted that high CI level originating from “random order,” i.e., an order (or schedule) consisting of the trials arranged randomly and rapidly changing, hindered performance and facilitated retention and transfer. On the other hand, the so-called “blocked order,” i.e., an order in which one skill variation trials were completed before introducing the next skill, resulted in a low CI level. Low CI, unlike high CI, facilitated performance and hindered retention and transfer.

This rather unexpected finding was soon tested in motor learning, originally by Shea and Morgan (1979) and later by many others. In Brady (2004) meta-analytically studied CI effect in motor learning concluding that the general (on retention and transfer) effect size (ES) in laboratory research was medium (Cohen’s *d* = 0.57) and in applied research was negligible (Cohen’s *d* = 0.19). On the other hand, the ES in studies on transfer in adults was small, i.e., *d* = 0.47 for laboratory research and *d* = 0.43 for applied research. In children, the ES in applied research was negligible (*d* = 0.10).

Retention and transfer, though both equally important in motor learning measure assess different constructs. Retention is a good indicator of learning, whereas transfer is a good indicator of adaptability (Magill and Anderson, 2021). One could argue that the ultimate goal of practice is to transfer skills from one context to another, i.e., generalize practiced skill to a non-practice one (Raviv et al., 2022). Consequently, transfer could be considered more reliable measure of CI effect (Shewokis, 1997; Brady, 2004). It is hence crucial to analyze and discuss CI effect on transfer exclusively.

Almost 20 years have passed since Brady performed his meta-analysis. Given that the meta-analysis should be updated within 2 years (Cumpston and Chandler, 2019) and the median time for the update in Cochrane Collaboration is about 5.5 (Shojania et al., 2007), the meta-analysis on the CI effect should have been updated much earlier. Some other analyses were performed in the meantime. However, most of them focused on very specific questions, e.g., CI effect in random vs. serial practice (Lage et al., 2021), CI effect in children with cerebral palsy (Graser et al., 2019), or CI effect in students in medical and physiotherapy education (Sattelmayer et al., 2016).

Recently, in 2023, Ammar and colleagues published a meta-analysis (Ammar et al., 2023) on the CI effect on retention and transfer in sport settings. They referred to the CI effects as “a myth” because they found statistically non-significant and small effect sizes favoring random practice in transfer and retention tests (in analyses on the whole population). Unfortunately, they did not report how they treated outcomes retrieved from one sample (multiple dependent effect sizes), i.e., how they dealt with the dependency problem. Additionally, they did not screen the EBSCO databases, which include key resources like APA PsycArticles, APA PsychInfo, SPORTDiscus with Full Text, Medline, and Academic Search Complete. Furthermore, they did not preregister their study, a now common practice in research that reduces publication bias and selective reporting of outcome-related bias.

Given all of the above, we decided to perform a systematic review, and we formulated our objectives based on the ones advanced by Brady (2004). However, we exclusively focused on transfer due to the number of conducted analyses and included studies. The study’s primary objective is to determine the overall effect size of CI on transfer in motor learning. Our secondary objectives, based on Brady (2004) inclusion criteria, are:

1. To estimate the CI effect in laboratory vs. field-based studies
2. To estimate the CI effect in young vs. adults vs. elderly adults
3. To estimate the CI effect in novice vs. experienced participants

## Methods

The study was registered in PROSPERO under the number CRD42021228267. Given the number of conducted analyses and included studies as well as the page/word limit for an article, the registered review has been split into two consecutive papers: retention (Czyż et al., 2023) and transfer meta-analyses. Transfer performance was analyzed in the present study. Each consecutive step of the study was performed in accordance with the PICO guidelines (Methley et al., 2014), the PRISMA statement (Moher et al., 2009, 2015; Page and Moher, 2017; see Supplementary Appendix 1), and, successively *Quality Assessment Tool for Quantitative Studies* (Thomas, 2003; Thomas et al., 2004).

### Inclusion and exclusion criteria

The inclusion criteria were defined in accordance with PICO (*Population*, *Intervention*, *Control*, *Outcome*) and included:

*Population:* adult, young, novice, experienced. In line with Brady’s inclusion criteria (Brady, 2004), only healthy participants were considered, and two variables (age and experience) were incorporated. Participants under 18 years old were labeled as *Young.* Participants aged 60 years old and more were classified as *Older Adults,* and those between 18 and 60 years old were ranked as *Adults.*

*Intervention*: field setting, high CI volume (random/interleaved schedule);

*Control*: laboratory settings, low CI volume (blocked schedule/repetitive practice);

A wide variety of motor tasks and experimental procedures were utilized in reviewed studies potentially considered for inclusion; however, it is worth mentioning that only the studies using single-task procedures were relevant for the current review.

The main classification considered for analysis was based on contextual interference volume: studies including groups with different practice schedules: blocked order (low CI), and random order (high CI) were compared.

The subgroup analysis was performed based on the age of participants: young (<18 years old), adults, and older adults (>60 years old). The subsequent subgroup analysis was the nature of the research: studies carried out in a controlled laboratory environment were matched up with studies conducted in a field setting using typical sports skills (applied research). The subsequent subgroup analysis was the experience—experienced participants vs. novices.

*Outcome*: transfer test results. The primary outcomes were the standardized effect sizes of CI in transfer in motor learning. The outcomes evaluating the transfer of the learned motor skill were considered selectable. Taking into account the effect of *sleep-induced consolidation of trained skills* (Diekelmann and Born, 2010; Yang et al., 2014) current meta-analysis consisted of delayed-transfer results. Studies covering immediate-transfer testing were discussed in the systematic review. This approach is similar to Brady (2004) since he used only delayed outcomes, assuming that learning effects may be obscured in the more immediate measures.

### Search methods and selection procedure

AW performed the search on the following databases: EBSCO (“contextual interference” in Title OR Abstract—no Keywords option), Scopus (“contextual interference” in Title OR Abstract OR Keywords), ScienceDirect (“contextual interference” in Title OR Abstract OR Keywords), Web of Science (“contextual interference” in Topic), in September 2021 (2020 to 2021), and updated March 2022 (period 2021–2022) and November 2022. Relevant studies were scrutinized using the Google Scholar search engine (“contextual interference” in Title OR Abstract OR Keywords). SC searched the PsycINFO database (“contextual interference” in Title OR Abstract OR Keywords).

Studies in languages other than English and the “gray literature” (e.g., master and Ph.D. dissertations and theses, conference proceedings, non-reviewed articles, etc.) available online in the searched databases were excluded to facilitate the reliable risk-of-bias assessment. Exclusion of non-English literature does not cause systematic bias (Morrison et al., 2012) and the proper and reliable translation from other languages into English may be problematic (Jackson and Kuriyama, 2019). Moreover, Jackson and Kuriyama noticed that only 2% of the articles included in the systematic reviews were published in languages other than English (Jackson and Kuriyama, 2019). On the other hand, there was no reason to include gray literature, since the topic (CI) is not novel (Gunnell et al., 2020) and gray literature inclusion would cause unnecessary burden on the reviewers (Mahood et al., 2014). Some of the non-peer-reviewed documents have different structure, different length, some of them are not peer-reviewed at all, some only partially.

Due to a large number of retrieved studies, a method proposed by Dundar and Fleeman (2017) was applied, i.e., AW evaluated the titles, abstract, and keywords for inclusion criteria, and a random sample was cross-checked by the senior researcher (SC). Inadequate articles were excluded, and duplicates of identified studies were removed. Subsequently, the full text of each study was read by the two co-authors (AW and PS), who independently assessed the papers for final eligibility. In case of a disagreement, a senior researcher (SC) was consulted, and a consensus was reached.

### Data collection and analysis

During the screening process, all relevant data were summarized by AW and PS in developed MS Excel data extraction forms. Each entry consisted of study specifications, such as the authors’ names, the study title, the year of publishing, and the journal title, in case of multiple experiments in the same study—number of experiments. The following details were based on PICO criteria:

- *Population* (age, gender, number of participants, expertise level).
- *Intervention* (testing procedure, dependent variable, nature of research, practice schedule, type of motor task).
- *Objectives/Outcomes* (immediate transfer results and delayed transfer results: extracted means and standard deviations for all groups and all measures). Consistently with our meta-analysis on retention (2023?), the results of the first block only from the transfer testing procedures were considered for the extraction. We assumed that the following blocks might enhance further learning. If the standard error of the mean (SEM) was available, we converted it into standard deviation (SD). If quartiles were available, we used the Mean Variance calculator (Luo et al., 2018; Shi et al., 2020a,b) to convert these into SD. Furthermore, study quality indicators were included, covering the following sections: selection bias, study design, confounders, blinding, data collection methods, withdrawals and dropouts, and global rating, based on *Quality Assessment tool for Quantitative studies* (Thomas, 2003; Thomas et al., 2004).

Since included studies utilized different motor skills (tasks) and the transfer was measured with different units (meters, seconds, number of cycles etc.) and scores (percentages, numbers), we used standardized mean differences (SMDs) effect sizes, i.e., Hedges’ (adjusted) g, very similar to Cohen’s d, but it includes an adjustment for small sample bias. The *I*^2^ statistic was used to evaluate the heterogeneity among the studies. The interpretation of *I*^2^ is as follows: 30–60% represent moderate heterogeneity; 50–90%—substantial heterogeneity; and 75–100%—considerable heterogeneity (Higgins et al., 2021). Nevertheless, interpretation thresholds can be misleading (Deeks et al., 2019).

In line with the Cohen’s recommendation of interpreting the magnitude of SMD in the social sciences (Cohen, 1988), we applied the following guidelines: small (SMD = 0.2); medium (SMD = 0.5); and large (SMD = 0.8).

We computed a 3-level mixed model which uses (restricted) maximum likelihood procedures (Cheung, 2014; Assink and Wibbelink, 2016). The advantage of that model is that it takes into account the potential dependence among the effect sizes, i.e., when there are multiple outcomes (effect sizes) from a single study. The model assumes that the random effects at different levels and the sampling error are independent. Three levels of the model refer to variance between effects sizes among participants (level 1), outcomes, i.e., effect sizes extracted from the same study (level 2; within-cluster variance), and studies (level 3; between-clusters variance) (Assink and Wibbelink, 2016). There is no need to know the correlations between outcomes extracted from one study for estimating the covariance matrix of the effect sizes since the second level in the model accounts for sampling covariation (Assink and Wibbelink, 2016).

Sensitivity analysis was performed using Cook’s D distances. Outcomes further than 4/n (where n was the number of outcomes) were removed to assess how these outliers influence the pooled effect.

Meta-analyses were performed with RStudio (version 2023.06.0) and the following packages “metaphor,” “dmetar,” “tidyverse,” “readxl,” and “ggplot.”

### Assessment of risk of bias/quality assessment of included studies

We followed the guidelines of the Effective Public Health Practice Project (EPHPP) *Quality Assessment Tool for Quantitative Studies* (Thomas, 2003) while assessing the risk of bias in included studies. The checklist elements (sample selection, study design, identification of confounders, blinding, reliability and validity of data collection methods, withdrawals, and dropouts) could be rated strong, moderate, or weak. According to a standardized guide and dictionary, the comprehensive evaluation is determined by assessing six rating aspects. Studies with two or more weak ratings are considered weak, those with less than four strong ratings and one weak rating are considered moderate, and, subsequently, studies with no weak ratings and at least four strong ratings are regarded as strong (Thomas et al., 2004). AW and PS independently assessed the level of evidence and methodological quality of the eligible studies. In case of discrepancy, the authors discussed until a consensus was reached. Any issues regarding the quality of the study was discussed with the senior researcher (SC).

## Results

### Results of the search

**Figure 1:**
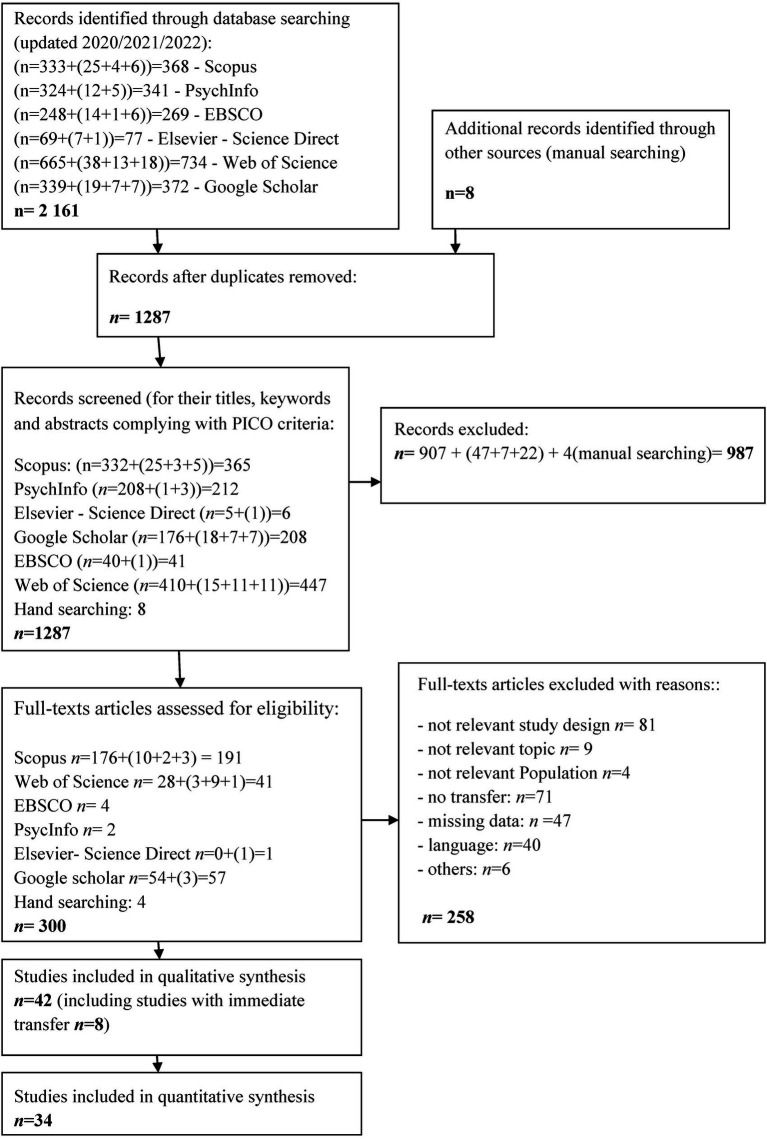
PRISMA flow diagram of the search process (Moher et al., 2009). Flowchart of the primary search (1966–2020), updated searches (2020 to 2021 and 2021 to 2023), and the inclusion and exclusion process.

The primary search in the databases identified 2,161 potential records. After removing duplicates, the titles and abstracts of 1,287 articles were screened according to PICO criteria, including 8 records identified manually (see Supplementary Appendix 2 for more details). Nine hundred eighty-seven articles (987) were excluded due to study design issues, not relevant topics, and population. The detailed evaluation process is highlighted in the PRISMA Flow Diagram (Moher et al., 2009; Figure 1).

The remaining 300 studies were evaluated, and 258 were excluded (comprehensive reasons for exclusion are listed in Supplementary Appendix 3). In case of missing data, the authors were contacted via e-mail and/or the ResearchGate platform. Finally, 42 articles were included in the present systematic review. The quantitative analysis covered 34 studies. Transfer tests conducted up to 24 h after the acquisition were defined as *immediate transfer testing,* and consequently, testing procedures performed after 24 h were defined as a *delayed transfer*. Eight studies described immediate transfer testing; therefore, the meta-analysis did not include these results. The summary of all included studies is provided in Supplementary Table S1. The summary of studies characteristics is presented in Supplementary Table S2.

### Reasons for exclusion of individual experiments or particular groups of participants

Occasionally, more than one experiment was presented in a single paper. There were cases where the authors did not report data on all of them, or, similarly, some studies did not report statistically non-significant data on specific variables. In such situations, we contacted authors; however, the authors did not provide the data in a few cases—the main reported reason was that their studies were performed a long time ago.

An article by Ste-Marie et al. (2010) consisted of three experiments where the authors examined the CI effect on handwriting skills in young participants. Unfortunately, it was not possible to obtain data from the first experiment. In the second experiment, data on the scores measure, and in the third experiment, data on the time variable were unavailable. In the paper by Porter and Magill (2010) covering two experiments, the results of the first one were available. The second experiment was excluded from the analysis due to group characteristics not compliant with PICO: group of ratio-feedback and segment-feedback. In the study by Chua et al. (2019) three experiments were conducted; however, the results of the first one were excluded from our review as these were describing constant practice group instead of blocked practice.

Some of the included studies covered more than two (random and blocked) groups of participants. In line with PICO criteria, in that situation, only the results of groups with blocked and random/interleaved schedules were regarded as appropriate for the analysis. Included primary studies consisting of more than two groups are listed below. In the study by Goodwin and Meeuwsen (1996), participants were randomly assigned to three groups: random, blocked/random, and blocked. Transfer results of blocked and random groups were included in the meta-analysis. Participants of the study by Porter and Beckerman (2016) were randomly allocated to three groups with random, increasing, or blocked schedules. The random and blocked group transfer data were considered applicable for the current analysis. In the study by Beik et al. (2021), participants were randomly assigned into six groups of blocked-similar, algorithm-similar, random-similar, random-dissimilar, blocked-dissimilar, and algorithm-dissimilar. Out of these groups, transfer results of four (blocked-similar, blocked-dissimilar, random-similar, random-dissimilar) were considered appropriate according to PICO. A similar situation occurred in the study by Beik and Fazeli (2021), where participants were randomly allocated to one of six groups of blocked-similar, blocked-dissimilar, learner-adapted-similar, random-similar, random-dissimilar and learner-adapted-dissimilar. Four groups were considered appropriate for analysis (random-similar, random-dissimilar, blocked-similar, blocked-dissimilar).

### Results of quality assessment of included studies

The detailed methodological assessment of the included studies is presented in Supplementary Appendix 4. None of the included studies was assessed as strong. Only two articles (Ste-Marie et al., 2010; Johnson et al., 2022) presented moderate methodological quality according to the *Quality Assessment Tool for Quantitative studies* (Thomas, 2003). The primary studies failed mainly on the latter criteria: 26 studies scored weak in Selection Bias, more than 21 articles scored weak in the *Confounders* section, 30 received weak ratings in the *Withdrawals and dropouts* section. The applied assessment tool specification might explain such relatively strict evaluation: two weak ratings were enough to automatically determine a weak classification of a study in its global rating for all six determinants of the checklist.

According to Thomas et al. (2004), only articles rated as moderate or strong should be included in the meta-analysis. Nevertheless, excluding all articles with *weak* global ratings would make the current analysis relatively doubtful (with only two studies included). For this reason, we have decided to include 34 studies in the meta-analysis. The impact of this decision on heterogeneity was taken into account.

### Findings

As aforementioned, only delayed transfer results were considered for the current meta-analysis, yielding 86 effect sizes. Outcomes of 34 studies were included, resulting in testing 1,421 participants. A wide range of variables was involved: time (absolute error time, decision time, variable time, response time, reaction time, completion time), the number of performed movements, distance (absolute error distance, accuracy error distance, median pathway traveled), accuracy (proficiency percentage, accuracy scores). Evaluation of transfer was presented by the mean of various outcome measures’ units: meters, seconds, percentages, or scores.

Motor skills utilized in primary studies varied in many ways. They were presented in different arrangements: discrete and continuous motor skills, open and closed motor skills, or fine and gross motor skills. Motor tasks were associated with volleyball (Bortoli et al., 1992; Meira and Tani, 2003; Fialho et al., 2006; Travlos, 2010; Pasand et al., 2016), golf (Goodwin and Meeuwsen, 1996; Porter and Magill, 2010; Chua et al., 2019), tennis (Broadbent et al., 2015), hockey (Cheong et al., 2016), throwing darts (Meira and Tani, 2001; Frömer et al., 2016), hopping (Parab et al., 2018), basketball (Porter et al., 2020), baseball (Hall et al., 1994), throwing (Vera and Montilla, 2003; Chua et al., 2019), learning of the Pawlata roll (Smith and Davies, 2008) and riffle shooting (Moretto et al., 2018). Non-sports skills included laparoscopic skills (Shewokis et al., 2017; Johnson et al., 2022) and handwriting (Ste-Marie et al., 2010), and Wii-Fit dynamic balance task (Jeon et al., 2020).

The other motor tasks applied in the primary studies covered: serial reaction time tasks (Lin et al., 2018), key pressing tasks (Del Rey et al., 1994; Shewokis, 1997; Shea et al., 2001; Meira et al., 2015; Beik et al., 2021), pursuit tracking tasks (Dunham et al., 1991; Porter and Beckerman, 2016), positioning tasks (Perez et al., 2005; Lage et al., 2006) and reversal or rapid movements on manipulandum (Green and Sherwood, 2000; Herzog et al., 2022).

### Laboratory versus applied setting—comparison characteristics

Laboratory vs. applied meta-analytic comparison included 34 studies. Seventeen studies, covering 46 effect sizes in total, were describing experiments conducted in laboratory settings. There were 738 participants in laboratory studies, of which 121 were older adults, and 542 were adults. The age of participants in the aforementioned studies ranged from 10 ± 0.6 years (Perez et al., 2005) to 82 years (Jeon et al., 2020). It is worth mentioning that only 75 participants from the laboratory studies were less than 18 years old (Perez et al., 2005; Broadbent et al., 2015). Motor skills utilized in the laboratory setting were: Wii-Fit dynamic balance task (Jeon et al., 2020), rapid movements on manipulandum (Green and Sherwood, 2000; Herzog et al., 2022), pursuit tracking tasks (Dunham et al., 1991; Porter and Beckerman, 2016), key pressing tasks (Shewokis, 1997; Shea et al., 2001; Meira et al., 2015; Beik et al., 2021), serial reaction time task (Lin et al., 2018), positioning tasks (Perez et al., 2005; Lage et al., 2006). In the study of Broadbent (Broadbent et al., 2015), the acquisition of tennis skills was conducted in laboratory settings—similar to the study by Chua et al. (2019), where the acquisition and testing of throwing skills and golf were assessed in the laboratory environment. An article by Frömer et al. (2016) described virtual darts throwing in the laboratory environment.

Applied studies were performed in natural environments (during physical education classes or game-based). Seventeen articles described experiments conducted in an applied setting, covering 40 effect sizes. Six hundred eighty-three (683) participants were included in these studies, of which 393 were under 18 years old, and 290 were adults. An article by De Souza et al. (2015) was the only study describing the transfer of motor skills in applied settings in older adults (65–80 years old). The motor task implemented in the study consisted of throwing a boccia ball to three targets. However, this study was excluded from the meta-analytic analysis due to missing data. The age of participants in the aforementioned studies ranged from 5.5 years (Ste-Marie et al., 2010) to 35 years (Thomas et al., 2021).

The following motor skills were practiced and examined in the included applied studies: golf skills (Goodwin and Meeuwsen, 1996; Porter and Saemi, 2010), volleyball skills (Bortoli et al., 1992; Meira and Tani, 2003; Fialho et al., 2006; Travlos, 2010; Pasand et al., 2016), hockey (Cheong et al., 2016), baseball (Hall et al., 1994), throwing skills (Vera and Montilla, 2003), riffle shooting (Moretto et al., 2018). In the study by Moretto et al. (2018) acquisition and testing of riffle shooting were conducted in indoor laboratories; however, all adjustments, including the position target height, followed the *Olympic and International Shooting Sport Federation* standards; and for this reason results were included in the *applied setting* comparison. Pawlata roll (Smith and Davies, 2008) learning and testing procedures took place in the indoor pool. CI effect on handwriting skills in children was tested by Ste-Marie et al. (2010) in the school setting. Laparoscopic skills in the study by Shewokis et al. (2017) were performed by medical students and post-graduate residents using a virtual reality simulator (LapSim^®^ VR simulator), mimicking the regular laparoscopic tasks. In the study by Johnson and colleagues (Johnson et al., 2022), laparoscopic skills were practiced and tested with the use of *Fundamentals of Laparoscopic Surgery* (FLS) box trainer (VTI Medical, Waltham, MA). The laparoscopic tasks were acquired and tested in both articles according to the FLS program.

### The CI effect in youth vs. adults vs. elderly adults—comparison characteristics

The age of participants in the studies included in the present review ranged from 5.5 years (Ste-Marie et al., 2010) to 82 years (Jeon et al., 2020). The age subgroup analyses were classified as follows: young (up to 18 years old), adults (18 years old to 59 years old), and older adults (60 years and older). The eight articles reporting immediate transfer covered the results of 80 children (Del Rey et al., 1983a), 10 adolescents (Fialho et al., 2006), and 318 adults (Del Rey et al., 1983b; Smith and Rudisill, 1993; Del Rey et al., 1994; Meira and Tani, 2001; Lage et al., 2006; Sherwood and Duffel, 2010). In the delayed transfer studies, there were 468 young participants, 812 adults, and 121 older adults. The results of 20 participants aged 17–21 described in the article by Hall and colleagues (Hall et al., 1994) were not classified in any of the age subgroups analysis. According to specific age group criteria applied in the present study: young (up to 18 years old), adults (18 years old to 59 years old), and older adults (60 years and older), the participants from the aforementioned study shall be included in both groups young and adults simultaneously. Therefore, we refrained from including their results.

### The CI effect in novice vs. experienced participants—comparison characteristics

In his meta-analytic study on CI, Brady (2004), among the other subgroup analyses, compared the CI effect between novice and skilled participants. While classifying their skill levels, Brady was guided by how the authors labeled the participants’ experience. We utilized the same rule in our review. In the immediate transfer articles, 90 participants were described as skilled (Del Rey et al., 1983a; Fialho et al., 2006). Out of the studies on delayed transfer, we classified 62 participants as experienced (Hall et al., 1994; Broadbent et al., 2015; Porter et al., 2020). Participants of these studies were characterized as follows: “*intermediate-level junior tennis players*” (Broadbent et al., 2015, p. 1245), “*baseball players from a junior college baseball team (…) with an average of 9.5 years of experience in competitive baseball”* (Hall et al., 1994, p. 837–838), participants “*had less than two years’ basketball playing experience (1.1 ± 1.3 years) and no representative level basketball playing experience*” (Porter et al., 2020, p. 7).

The primary studies in the current review referred to different inclusion standards for a *skilled* group. Classifying skill (experience) levels correctly could lead our review in a different direction, still not warranting there will be no confusion or doubts. Therefore, we decided not to conduct the subgroup analysis of *skilled* versus *novice*.

## Meta-analysis: results

**Figure 2:**
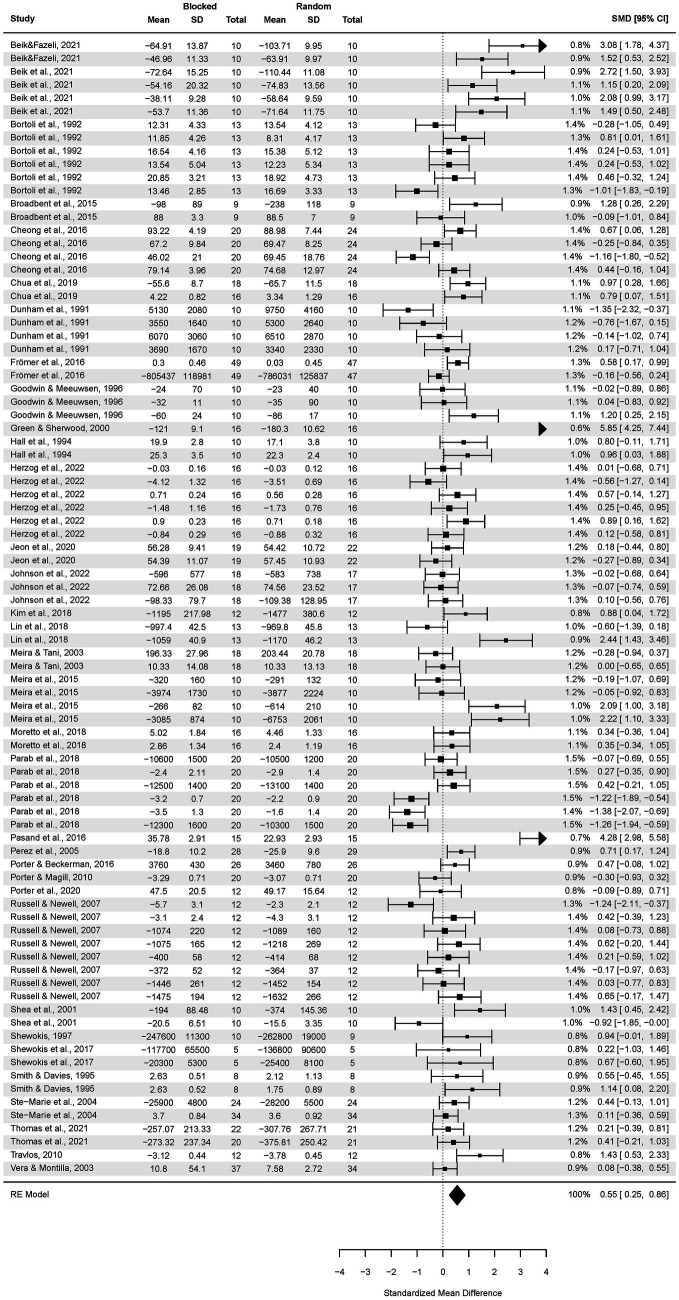
Analysis of transfer test results of random practice vs. blocked practice. The forest plot presents the results obtained by participants aged 5.5–82, including various motor tasks and different outcome measures.

The analysis of the CI effect on delayed transfer (Figure 2) covered 34 studies, yielding 86 effect sizes and resulting in testing 1,421 participants.

The pooled effect size based on the three-level meta-analytic model was medium SMD = 0.55 (95% CI: 0.25, 0.86; *p* < 0.001). The estimated variance components (tau squared) were τ_3_^2^ = 0.488 and τ_2_^2^ = 0.403 for the level 3 and level 2 components, respectively. This means that *I^2^_3_* = 47% of the total variation can be attributed to between-cluster, and *I^2^_2_* = 39% to within-cluster heterogeneity. Total *I^2^* = 86%. We found that the three-level model provided a significantly better fit compared to a two-level model with level 3 heterogeneity constrained to zero (χ^2^ = 9.99; p < 0.001): for three level model (df = 3) AIC = 246 while for the 2-level model AIC = 254. Test of moderators: F(1, 84) = 1.34, *p* = 0.25 suggesting the subgroup analyses are not necessary, though, we decided to perform them analogically to Brady (2004).

**Figure 3:**
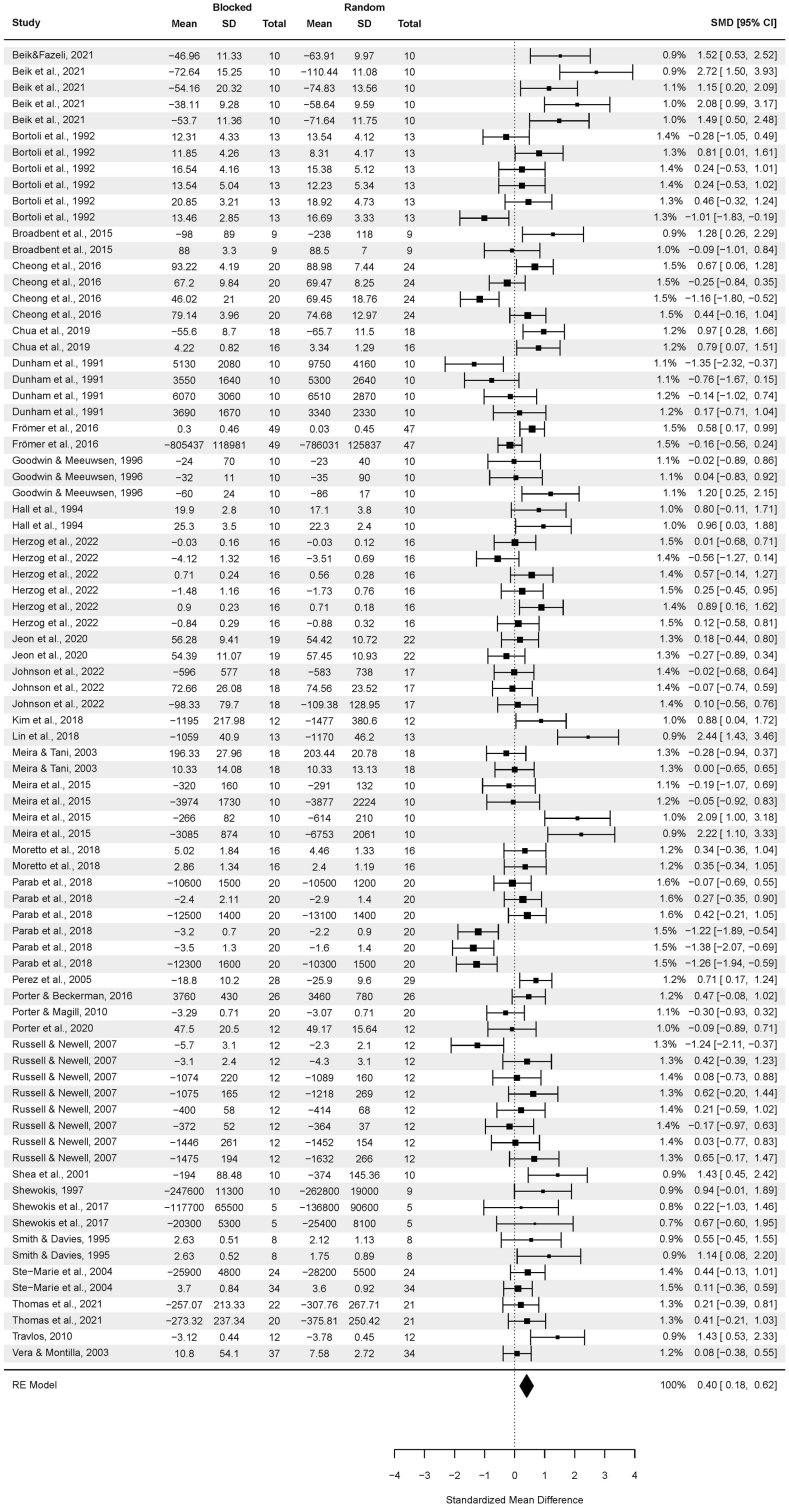
Analysis of transfer test results of random practice vs. blocked practice with no outliers (further than 4/n Cook D distances).

Sensitivity analysis revealed that there were 5 outcomes which were further than 4/n threshold (Figure 2). These were outcomes from Beik et al. (2021) and Beik and Fazeli (2021)—one outcome, Green and Sherwood (2000) and Parab et al. (2018). After having removed the outliers (Figure 3), the pooled effect size was small SMD = 0.40 (95% CI: 0.18, 0.62; *p* < 0.001). The estimated variance components (tau squared) were τ^2^_3_ = 0.19 and τ^2^_2_ = 0.23; *I^2^_3_* = 34% and *I^2^_2_* = 41%; respectively. The outliers had a substantial effect on pooled effect size, i.e., SMD decreased from 0.55 (with outliers included) to 0.40 (without outliers).

### Laboratory vs. field-based (applied) studies

The primary studies were divided into those carried out in a laboratory setting (*n* = 17), including 738 participants (46 effect sizes), and the remaining (*n* = 17) conducted in an applied setting (40 effect sizes), including 683 participants.

**Figure 4:**
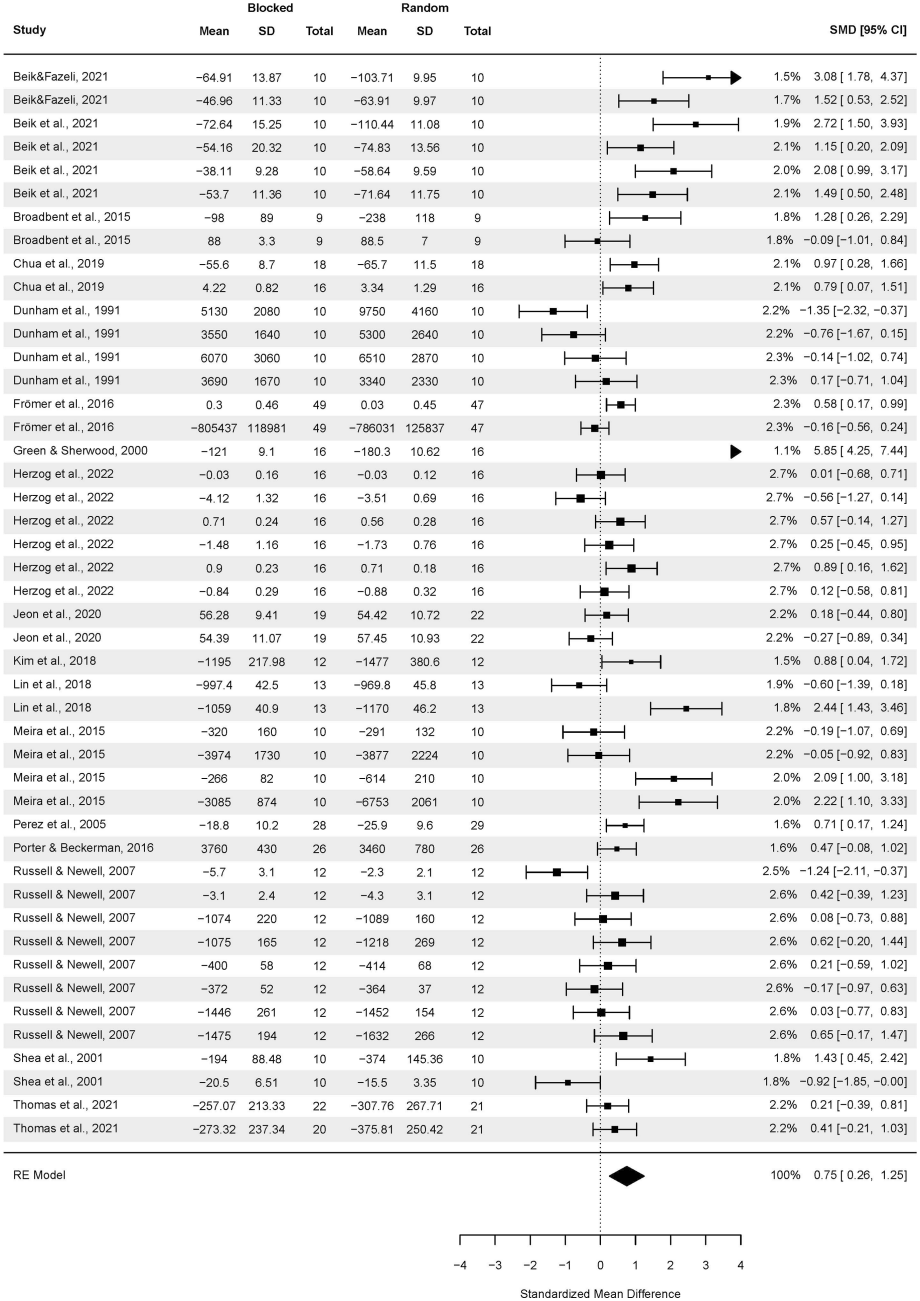
Analysis of transfer test results in a random and blocked schedule in a laboratory setting. The forest plot presents the transfer test results obtained by participants practicing in a laboratory setting, including various motor tasks and different outcome measures.

A subgroup analysis of the CI effect in laboratory studies was performed (Figure 4). The pooled effect size based on the three-level meta-analytic model was medium SMD = 0.75 (95% CI: 0.26, 1.25; *p* = 0.004). The estimated variance components (tau squared) were τ_3_^2^ = 0.68 and τ_2_^2^ = 0.52 for the level 3 and level 2 components, respectively. Heterogeneity was high, *I^2^_3_* = 50% and *I^2^_2_* = 39%; total *I^2^* = 88.96%.

Sensitivity analysis revealed that after removing two outcomes, i.e., Green and Sherwood (2000) and Lin et al. (2018), the pooled effect size dropped SMD = 0.62 (95% CI: 0.24, 1.01; *p* = 0.002).

**Figure 5:**
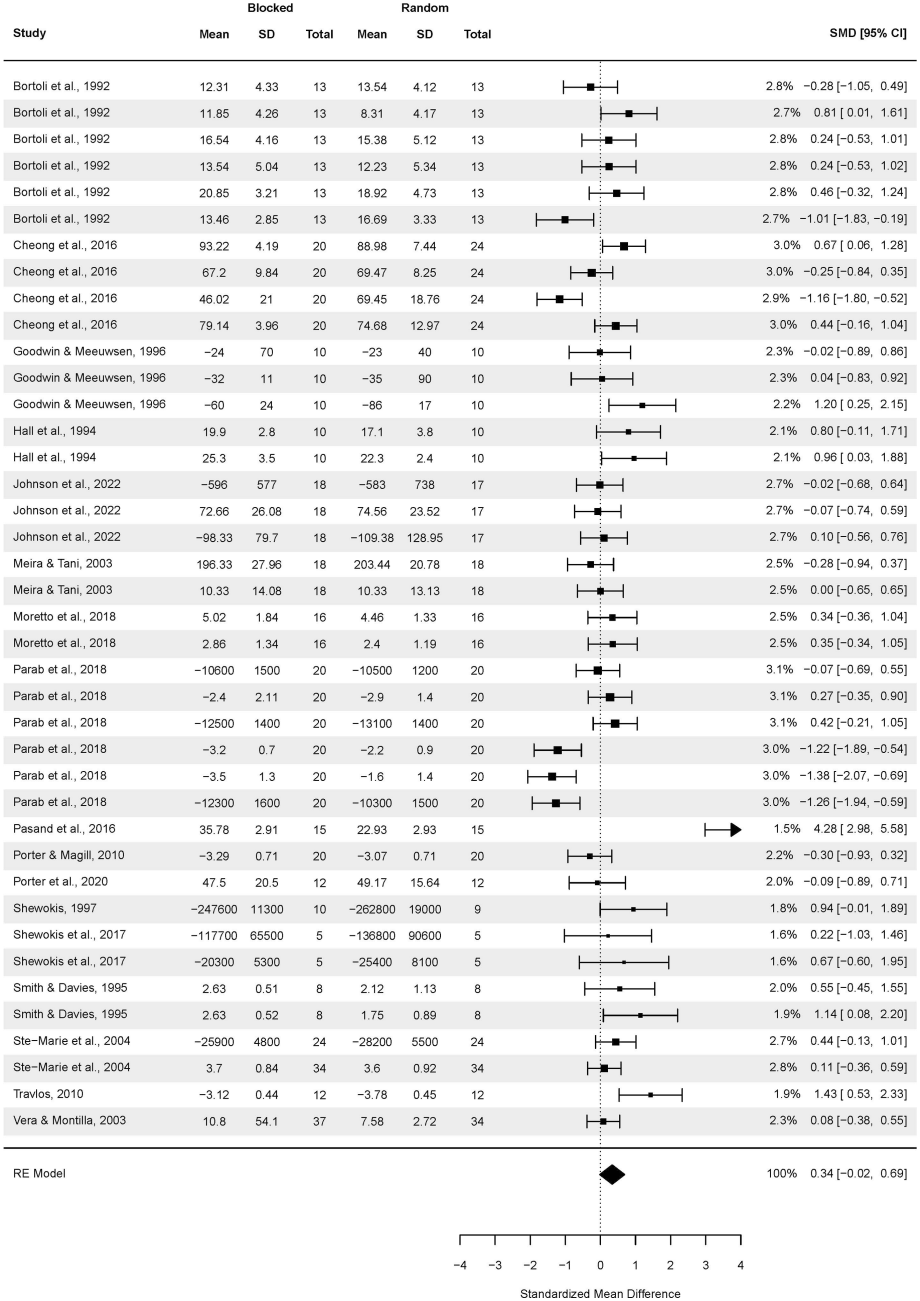
Analysis of random and blocked schedule transfer test results in an applied setting. The forest plot presents the transfer test results in an applied setting, including various motor tasks and different outcome measures.

Analogously, a subgroup analysis of the CI effect in applied studies was conducted (Figure 5). The pooled effect size based on the three-level meta-analytic model was small SMD = 0.37 (95% CI: −0.02, 0.69; *p* = 0.06). The estimated variance components (tau squared) were τ_3_^2^ = 0.27 and τ_2_^2^ = 0.31 for the level 3 and level 2 components, respectively. Heterogeneity was high, *I^2^_3_* = 37% and *I^2^_2_* = 44%; total *I^2^* = 81.35%.

Sensitivity analysis revealed that after removing two outcomes, i.e., Travlos (2010) and Pasand et al. (2016), the pooled effect size decreased to negligible SMD = 0.11 (95% CI: −0.13, 0.34; *p* = 0.36).

### The CI effect in young vs. adults vs. elderly adults

**Figure 6:**
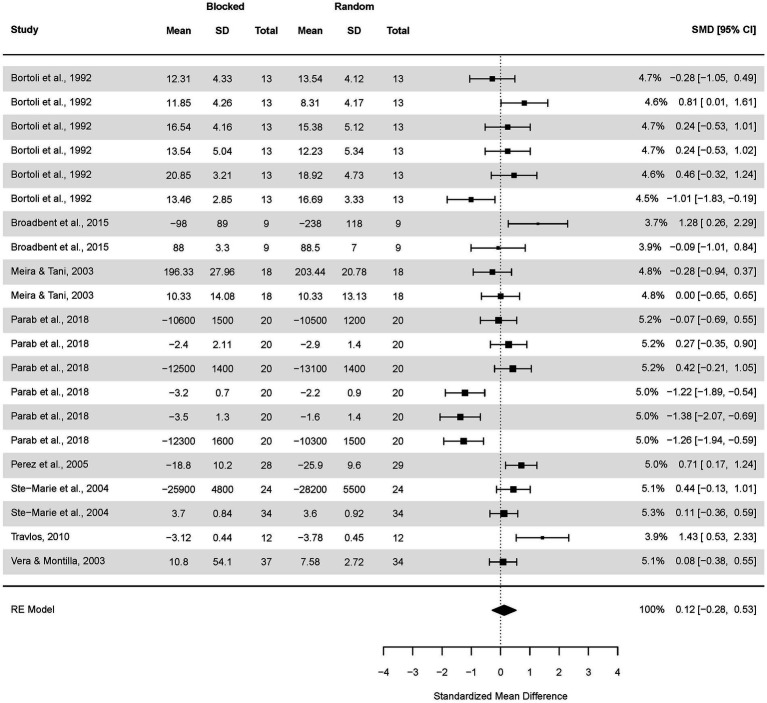
Analysis of young participants’ transfer test results: random vs. blocked practice. The forest plot presents the transfer test results of participants aged 5.5–18, including various motor tasks and different outcome measures.

Thirty-three articles were included in a meta-analytic comparison of the CI effect in three age groups (Figure 6), resulting in the testing of 468 young participants, 812 adults, and 121 older adults. As aforementioned, the results of 20 participants aged 17–21 from the study by Hall et al. (1994) were not included in the current subgroup analysis. This analysis yielded 2,632 measurements in total and elicited: 21 effect sizes for young participants, 55 effect sizes for adults, and eight effect sizes for the group of older adults.

Firstly, we performed an analysis for the subgroups of young participants. The pooled effect size based on the three-level meta-analytic model was negligible SMD = 0.12 (95% CI: −0.28, 0.53; *p* = 0.54). The estimated variance components (tau squared) were τ_3_^2^ = 0.10 and τ_2_^2^ = 0.31 for the level 3 and level 2 components, respectively. Heterogeneity was high, *I*^2^_3_ = 18% and *I*^2^_2_ = 60%; total *I^2^* = 78.42%.

Sensitivity analysis revealed that after removing one outcome, i.e., Travlos (2010), the pooled effect size decreased to SMD = 0.02 (95% CI: −0.36, 0.39; *p* = 0.36).

**Figure 7:**
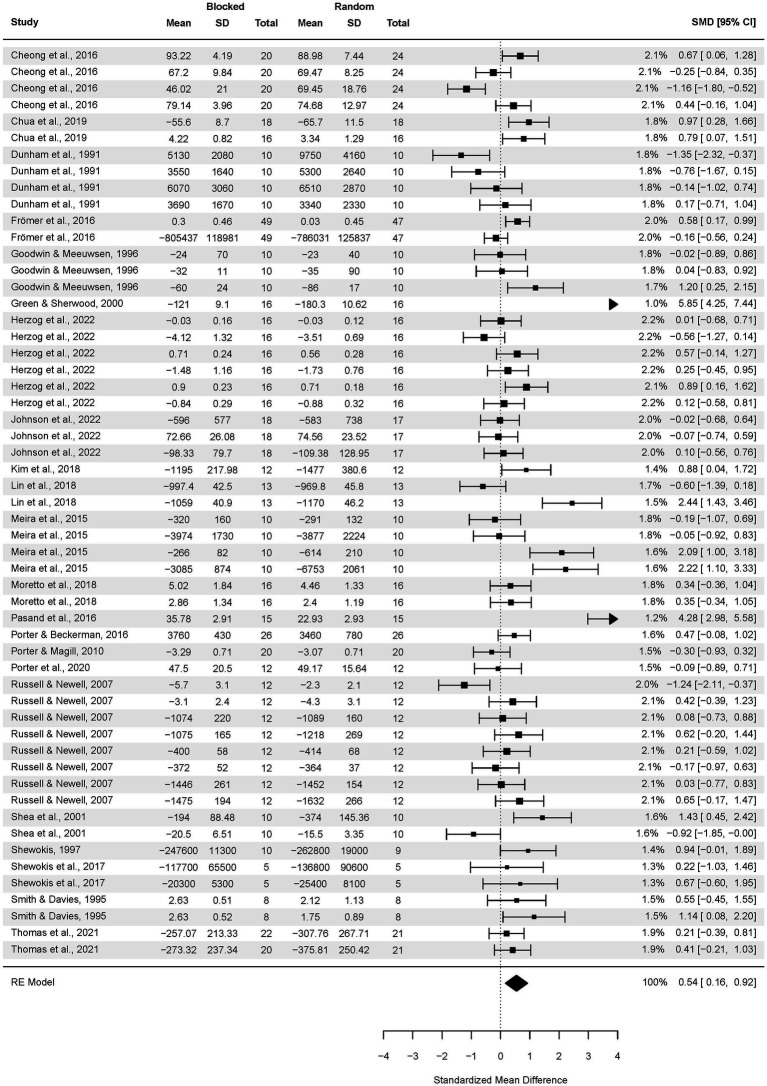
Analysis of adult participants’ transfer test results: random vs. blocked practice. The forest plot presents the transfer test results obtained by participants aged 18–59, including various motor tasks and different outcome measures.

Secondly, we analyzed the adult’s subgroup (Figure 7). The pooled effect size based on the three-level meta-analytic model was medium SMD = 0.54 (95% CI: 0.16, 0.92; *p* = 0.54). The estimated variance components (tau squared) were τ_3_^2^ = 0.42 and τ_2_^2^ = 0.53 for the level 3 and level 2 components, respectively. Heterogeneity was high, *I^2^_3_* = 39% and *I*^2^_2_ = 48%; total *I^2^* = 86.82%.

Sensitivity analysis revealed that after removing five outcomes, i.e., Green and Sherwood (2000), Shea et al. (2001), Travlos (2010), Pasand et al. (2016), and Lin et al. (2018), the pooled effect size decreased to medium SMD = 0.38 (95% CI: 0.13, 0.62; *p* = 0.003).

**Figure 8:**
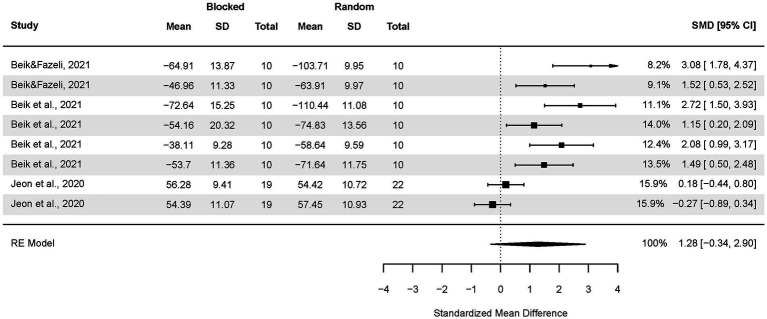
Analysis of older adults’ transfer tests results: random practice vs. blocked practice. The forest plot presents the transfer test results obtained by participants aged 60–82, including a variety of motor tasks and different outcome measures.

Thirdly, an analysis for older adults was performed (Figure 8). The pooled effect size based on the three-level meta-analytic model was large SMD = 1.28 (95% CI: −0.34, 2.90; *p* = 0.10). The estimated variance components (tau squared) were τ_3_^2^ = 1.24 and τ_2_^2^ = 0.15 for the level 3 and level 2 components, respectively. Heterogeneity was high, *I*^2^_3_ = 77% and *I*^2^_2_ = 10%; total *I*^2^ = 87.05%.

There were no outcomes further than 4/n Cook’s D distances.

### Risk of publication bias assessment

**Figure 9:**
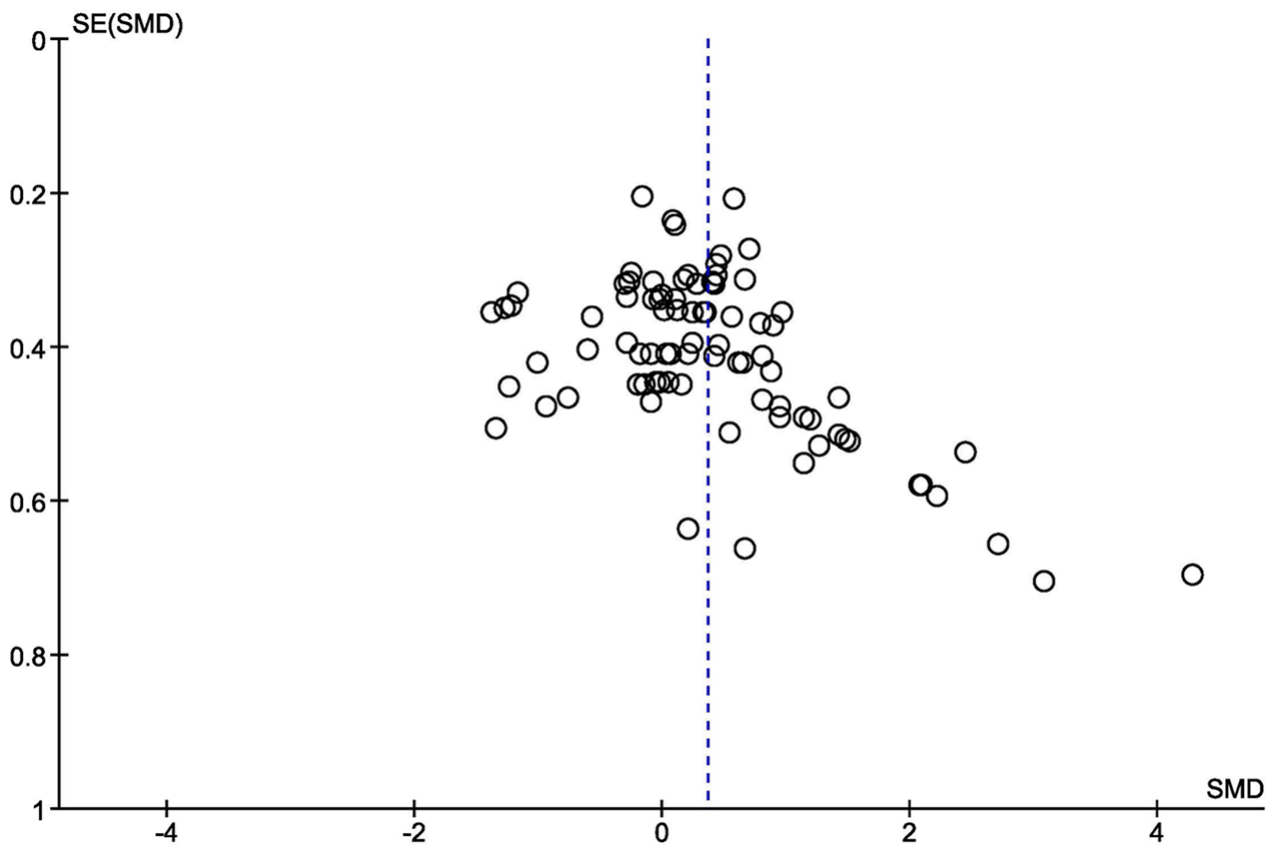
Risk of bias assessment—funnel plot.

The risk of publication bias was assessed using a funnel plot (Figure 9). Given that substantial heterogeneity was present in each of the analyses we performed, we decided not to apply other statistical methods, i.e., Egger’s regression test or rank-correlation test.

## Discussion

The study’s primary objective was to determine the overall effect size of CI on transfer in motor learning. We found that the statistically significant overall CI effect on transfer was medium (SMD = 0.55) in favor of the random practice.

Our secondary objectives were to estimate the CI effect on transfer in laboratory versus non-laboratory studies and the CI effect in different age groups. Similarly, to the overall effect, the random practice was favored in the laboratory and applied settings. However, in laboratory studies, the medium effect size was statistically significant (SMD = 0.75), whereas, in applied studies, the effect size was small and statistically non-significant (SMD = 0.34). Significant and larger effect sizes in laboratory settings may be due to well-controlled environmental variables and simpler tasks utilized in laboratories (Jeon et al., 2020). Complex tasks used in applied settings may be too challenging for information processing (Hebert et al., 1996) and deleterious for learning as a result (Wulf and Shea, 2002). However, there may be another explanation; as Al-Mustafa stated, CI is a laboratory artifact (Al-Mustafa, 1989; Brady, 2004), i.e., CI effect is conspicuous in labs but not in real life.

Age group analysis turned out to be significant only in adults and older adults. In both age categories, random practice was favored. In adults, the effect was medium (SMD = 0.54), whereas in older adults was large (SMD = 1.28). The results in the adult group align with Brady’s (Brady, 2004), who reported small effect sizes in both laboratory and applied settings. However, Brady did not recognize the older adult group; therefore, comparing the results with any previous ones is difficult. On the other hand, nonsignificant effect size in young participants was negligible (SMD = 0.12).

### Comparison with Brady’s and Ammar’s et al. meta-analysis

Only seven studies originally included in Brady’s meta-analysis, yielding 17 effect sizes, were included in our analysis. Unfortunately, data from 23 primary studies included in his meta-analysis were unavailable. On the other hand, 27 primary studies included in our meta-analysis (yielding 69 effect sizes) were not included in Brady’s.

The overall results of our review partially corresponded with those reported in the meta-analysis by Brady (2004). In line with the constantly advancing methodology of conducting meta-analyses, the inclusion criteria implemented in this review were more thoroughly detailed than those presented in Brady’s. Consequently, 13 following studies included in Brady’s meta-analysis (Brady, 2004) were considered irrelevant in the present review and, therefore, excluded. The primary studies by Wulf and Lee (1993), Sekiya et al. (1994, 1996), Landin and Hebert (1997), and Sekiya and Magill (2000) described serial practice order instead of random schedule.

In the studies by Wrisberg and Liu (1991), Hebert et al. (1996), Smith (2002), and Smith et al. (2003) alternating practice instead of a random schedule was presented. In the article by Hall and Magill (1995), an experiment described by Lee et al. (1992), and a study by Shea and Titzer (1993), multiple task learning was implemented. In the article by Bortoli et al. (2001), included in Brady’s meta-analytic study, constant and variable practice schedules were compared. Additionally, 15 studies did not include transfer tests. Three other studies described immediate transfer; therefore, our meta-analysis did not include the results.

Our results are different from those reported by Ammar et al. (2023). We found that the pooled effect size was medium (SMD = 0.55) and statistically significant, while Ammar et al. reported small (SMD = 0.243) and non-significant. In both studies, random practice was favored. Probably the differences between these studies may be attributed to the search strategies, number of studies and effects sizes included in both meta-analysis. Ammar et al. omitted EBSCO database (including APA PsycArticles, APA PsychInfo, SPORTDiscus with Full Text, Medline, and Academic Search Complete), specifying their focus differently (sport-specific). They finally included 16 studies and 38 ES referring to sport settings whereas in our meta-analysis 34 studies and 86 ES were included in general analysis and 17 studies and 40 ES in applied studies. Similarly, the differences in subgroups analysis can be explained in methods applied.

### Low quality and bias problem

The studies included in our analysis were of poor quality in general. None of the included studies was assessed as strong. Quality of two articles were assessed moderate. Hence, one could re-state our question if the studies about CI effect are biased? The question is justified given 26 studies scored weak on the *Selection Bias* criteria. One of the possible explanations of the week scores, in general, could be the tool we used, i.e., *Quality Assessment Tool for Quantitative Studies* (Thomas et al., 2004), which is rather strict. However, another explanation could be that the researchers’ bias toward specific results in laboratory setting affected the final effect size favoring random practice.

One of the possibilities to decrease the effect of low-quality studies the meta-analysis result would be to exclude all studies rated as *weak*. This is what Thomas and colleagues postulated (Thomas et al., 2004). However, it would leave our analysis with only two papers! Therefore, we decided to include all the studies, though it could have impacted the heterogeneity statistics, increasing the *I^2^* value.

### Heterogeneity problem

In all our analyses, the heterogeneity was substantial (with *I*^2^ > 70%). There may be a few explanations for why the heterogeneity was so high. Firstly, we included many studies and outcomes: more studies and outcomes, the higher the *I*^2^ value (Rücker et al., 2008). One could decrease the *I*^2^ value by limiting the number of studies included in the analysis and including only those with fewer participants (Schroll et al., 2011). However, this would question the validity of the presented review because a low *I*^2^ value is not necessarily linked with a lower probability of heterogeneity but may be linked to the lower sensibility of detecting it (Schroll et al., 2011). Alba et al. (2016) noted “*I^2^ can also mislead in large studies with precise results in which a low degree of inconsistency (*i.e.*, studies report similar point estimates) can nevertheless result in high I^2^*” (p. 134). They added that we are not able to do much about it. Moreover, there is little advice for the researchers on how to do it (Schroll et al., 2011).

Secondly, we included studies of low quality (see Low quality and bias problem section). Thirdly, the source of high heterogeneity may be linked with the differences in populations, such as age and origin, followed by a variety of included motor tasks and outcome measures or different methodologies used, e.g., experiment duration.

Lastly, we used *I*^2^-test to assess the heterogeneity level. Unlike the *Q*-test (used by Brady in 2004), *I*^2^ index not only informs about the presence or absence of heterogeneity but also quantifies its magnitude (Huedo-Medina et al., 2006).

## Limitations

Limitations of our analysis have to be acknowledged. Firstly, 34 included studies yielded 86 effect sizes. As a result, some of the studies contributed multiple effect sizes. We treated them as independent, similarly to Toth et al. (2020). Thus, the combined significance values have to be interpreted with caution since they may be inflated of combined probability level.

Secondly, we tried to update Brady’s meta-analysis (Brady, 2004), nevertheless, we failed at obtaining all results (outcomes) Brady included. These not-included results could affect the overall effect of our analysis.

Thirdly, we advanced our objectives based on Brady’s. One could perform more specific analyses (more specific PICOs) that could lead to different results.

### Recommendation for future research

Given that studies on CI effect on retention and transfer, were mostly of poor quality, a strong emphasis has to be put on the methodological issues.

## Data availability statement

The original contributions presented in the study are included in the article/Supplementary material, further inquiries can be directed to the corresponding author/s.

## Author contributions

SC: Conceptualization, Data curation, Formal analysis, Funding acquisition, Investigation, Methodology, Project administration, Resources, Software, Supervision, Validation, Visualization, Writing – original draft, Writing – review & editing. AW: Data curation, Formal analysis, Investigation, Resources, Validation, Writing – original draft, Writing – review & editing, Methodology. PS: Data curation, Investigation, Writing – original draft, Writing – review & editing.

## Supplementary material

All appendices are available at: https://osf.io/tqchf/?view_only=663162ef579347509b632b971b503eba

Supplementary Table S1 Summary of the included studies.

Supplementary Table S2 Characteristics of the tasks, participants, tests, and outcomes of the included studies.
